# Semantic modeling of cell damage prediction: a machine learning approach at human-level performance in dermatology

**DOI:** 10.1038/s41598-023-35370-7

**Published:** 2023-05-23

**Authors:** Patrick Wagner, Maximilian Springenberg, Marius Kröger, Rose K. C. Moritz, Johannes Schleusener, Martina C. Meinke, Jackie Ma

**Affiliations:** 1 Department of Artificial Intelligence, Fraunhofer Heinrich Hertz Institute, Einsteinufer 37, 10587 Berlin, Germany; 2 Department of Dermatology, Venereology and Allergology, Charité - Universitätsmedizin Berlin, Corporate Member of Freie Universität Berlin and Humboldt-Universität zu Berlin, Charitéplatz 1, 10117 Berlin, Germany

**Keywords:** Machine learning, Image processing, Computational models, Skin cancer

## Abstract

Machine learning is transforming the field of histopathology. Especially in classification related tasks, there have been many successful applications of deep learning already. Yet, in tasks that rely on regression and many niche applications, the domain lacks cohesive procedures that are adapted to the learning processes of neural networks. In this work, we investigate cell damage in whole slide images of the epidermis. A common way for pathologists to annotate a score, characterizing the degree of damage for these samples, is the ratio between healthy and unhealthy nuclei. The annotation procedure of these scores, however, is expensive and prone to be noisy among pathologists. We propose a new measure of damage, that is the total area of damage, relative to the total area of the epidermis. In this work, we present results of regression and segmentation models, predicting both scores on a curated and public dataset. We have acquired the dataset in collaborative efforts with medical professionals. Our study resulted in a comprehensive evaluation of the proposed damage metrics in the epidermis, with recommendations, emphasizing practical relevance for real world applications.

## Introduction

Skin cancer is one of the most frequent types of cancer and the success of a curative treatment depends strongly on the stage and a timely detection. The detection of skin cancer lesions is a diagnostic task that is performed by dermatologists and other medical professionals in medical institutions using different tools. The standard procedure is the examination by a dermatologist, using a dermatoscope, which can achieve high detection rates but is, however, subjective and strongly depends on the experience of the dermatologist. Depending on the suspected type of cancer common non-invasive procedures using laser-based microscopes can lead to accurate high detection rates with increased objectivity. Still, the most accurate (and costly) procedure involves a biopsy followed by a histological analysis. This procedure is indeed very time-consuming as the histopathologist has to analyse the histological slice on a cell basis. Histopathologists detect cells using patterns and morphology of cells^1^. Results of immunohistochemical stains are most commonly estimated in percent without counting a large number of cells (often 10 cells in a representative area). Therefore, Computer Aided Diagnostic (CAD) tools are becoming more and more useful in assisting medical professionals to improve the overall efficiency. Furthermore, technological advancements in Artificial Intelligence (AI), in particular in Deep Learning (DL), show great potential in improving image-based medical diagnosis even further^2–4^. In a study by Esteva et al. the authors have trained an end-to-end deep neural network that is able to classify skin cancer at a performance level that is comparable to dermatologists^5^. Subsequently, there have been many other studies and systematic reviews that analyse the potential and challenges of using AI in skin cancer detection on dermatoscopic images as well as histopathological images^6–9^.

The emergence and assessment of skin cancer can often be exposed by analysing the epidermis which is the outermost layer of the skin and therefore susceptible to skin cancer related influences such as sun exposure. The epidermis is build up by different types of cells such as Squamous cells, Basal cells, Melanocytes, and Keratinocytes that could potentially develop into cancer cells. As such, a highly accurate segmentation of the epidermal layer is often an important prerequisite for an automated analysis of whole slide images (WSI). This segmentation problem is part of ongoing research and is relevant for multiple imaging modalities such as histopathological images^10^ or Optical Coherence Tomography (OCT)^11^. In this work, we focus on the carcinogenic DNA damages caused by ultraviolet (UV) radiation on excised abdominal human skin, excised porcine skin and in vitro skin models with different melanin indices. Absorption of UV radiation produces two predominant types of DNA damage, cyclobutane pyrimidine dimers (CPD) and pyrimidine (6-4) pyrimidone photoproducts (6-4PP)^12,13^. CPD are the predominant UV-induced DNA lesions and are approximately five-fold more prevalent than 6-4PP. Both damages can be indicated by immunohistochemical staining. This is important for the comparison DNA damage induced by Far-UVC irradiation to that caused by daily sunlight exposure^14^. Since the damage is not always homogeneous, the evaluation of a representative small area is not sufficient.

**Figure 1:**
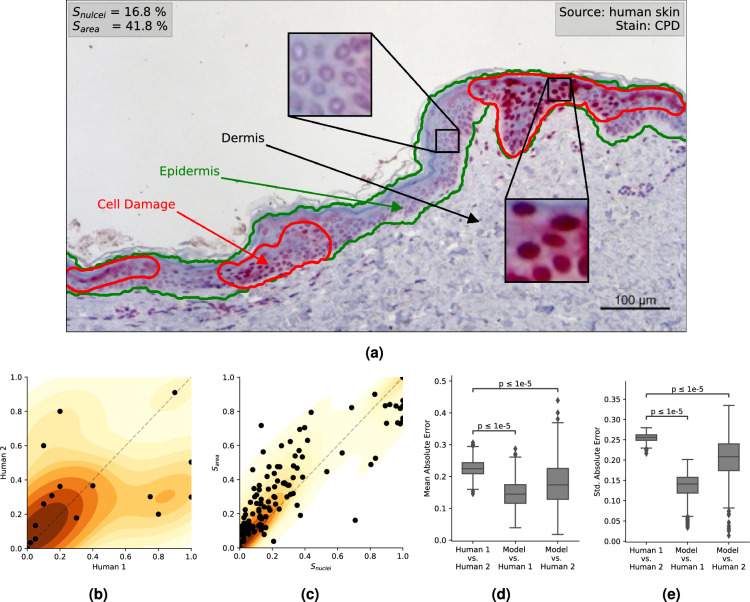
Depicted are an explanatory example of a histological slide with the red staining indicating CPD DNA-damaged nuclei of the keratinocytes in (**a**) and scatter plots of human-annotated relative cell-counts $S_\text {nuclei}$ and relative damaged area $S_\text {area}$ see (**c**). Underlaid is the kernel density estimation of the score value pairs. Yellow implies low density, red implies high density. (**b**) shows the correlation of two pathologists on 18 samples with heterogeneous damage. (**c**) shows the correlation of $S_\text {area}$ and $S_\text {nuclei}$ ground truth values. Underlaid is the kernel density estimation of the score value pairs.. For the analysis of human-level performance we compared the agreement of three scenarios: among (1) two human pathologists (2) the first pathologist and our regression model, (3) the second pathologist and our regression model. The results are visualized with respect to the mean of absolute errors (**d**), and standard deviation of absolute errors (**e**).

For the automation of this process we propose a data-driven approach, where we use machine learning methods for segmenting a whole slide image into three classes: (1) epidermis (2) damaged cells in the epidermis and (3) other cells and background. In Fig. 1a we give an example of a scanned skin section, where the epidermis (green) and the damaged cells (red) are annotated. In order to train models which are competitive with a human pathologist, we collected a dataset consisting of a wide variety of skin samples and labels. For this we obtained two kinds of labels: (1) a scalar with respect to the ratio of damaged cells ($S_\text {nuclei}$) and (2) pixel-wise annotations ($\textbf{Y}$) that infer a scalar score ($S_\text {area}$), obtained by a pathologist for a subset only (pixel-wise annotations are more expensive to obtain). The correlation of these scores is subjected to some noise, as they have been annotated by different pathologists. Sample noise occurs in samples with heterogeneous damage. For these critical samples, experts tend to conclude at different ratio of damaged to non-damaged cells, inducing noisy annotations of $S_\text {nuclei}$. This noise can be lessened when annotations from many pathologists are obtained and either averaged or a majority vote is performed to obtain final annotations. However, this process is very costly as many pathologists have to be consulted, in order to obtain reliable scores. We propose the $S_\text {area}$ score, which tends to yield less noise and correlates with $S_\text {nuclei}$ (see Fig. 1c). To assess human-level performance and associated sample noise, two pathologists individually labeled $S_\text {nuclei}$ of 18 heterogeneous samples. On these critical samples a mean absolute error (MAE) of 0.17 with a standard deviation of 0.22 was measured. In comparison, across all 202 segmented samples the $S_\text {area}$ score measures an MAE of 0.08 with a standard deviation of 0.11 and if we constrain the evaluation to critical samples where $0.1 \le S_\text {nuclei} \le 0.9$ we measure an MAE of 0.15 and a standard deviation of 0.12. Correlations of $S_\text {nuclei}$ with $S_\text {area}$ and the pathologists among another are visualized in Fig. 1b and c. We observe that $S_\text {area}$ generalizes well on $S_\text {nuclei}$, with a lower MAE than that of pathologists among another on critical samples. Though the annotation of semantic segments can be a little more time consuming than counting nuclei, the resulting labels appear to be more reliable and may justify the process.

## Results

**Table 1:** Results reported as mean and standard deviation of (10 fold) cross-validation with metrics specific for regression (mean absolute error MAE) and segmentation (Intersection over Union IoU and pixel-wise accuracy).$^1$ computed on all samples ($n=802$). $^2$ computed on samples with annotations ($n=202$). * used the area scores directly for comparing to nuclei score and vice versa.

Model	MAE $[S_{\text {nuclei}}]^1$ $\downarrow$	MAE $[S_{\text {area}}]^2$ $\downarrow$	IoU $[\textbf{Y}]$ $\uparrow$	Acc $[\textbf{Y}]$ $\uparrow$
Regression	${\textbf {0.075}} \pm {\textbf {0.003}}$	$0.078 \pm 0.003$*	$-$	$-$
Masked Regression	${\textbf {0.081}} \pm {\textbf {0.003}}$	$0.080 \pm 0.005$*	$-$	$-$
Segmentation	0.091 ± 0.002*	**0.044 ** ± **0.069**	0.785 ± 0.003	0.976 ± 0.001

Significant values are in [bold].

In Table 1 we report the results of our experiments as described in Material and Methods where we consider three different labels: (a) $S_{nuclei}$ (relative cell counts) (b) $S_{area}$ (relative areas derived from $\textbf{Y}$) and (c) the ground truth segmentation $\textbf{Y}$ of both (epidermis and damaged cells). For (a) and (b) we consider the mean absolute error (MAE) as an appropriate metric for regression tasks. We report the intersection over union (IoU) and pixel-wise accuracy (Acc) for the segmentation (c). We propose and evaluate three models: (1) plain regression model on the whole image regressing $S_{nuclei}$. (2) (Epidermis) masked regression model regressing $S_{nuclei}$ and (3) segmentation model predicting $\textbf{Y}$.

### Quantitative evaluation

Our evaluation features two kinds of regression models. One receiving the entire image (Regression) as input and one receiving a masked image (Masked Regression), where only the epidermis is visible as input. All masks have been received by a separately trained U-Net, that only segments the entire epidermis. We hypothesized that only the image-area where cell damage should be detected is relevant and that performance may be boosted if all other information is filtered out. However, global feature extraction may carry information of the staining, relative color saturation and other tissue-related characteristics of a sample. This information may also effect the decision procedure, even if it is not directly related to the score $S_\text {nuclei}$. We chose the pre-trained VGG 16 as the backbone model for the regression task. No clear benefits were observed when using more complex models, which may be due to the limited amount of data (we will elaborate more in the discussion). Overall, no gain in performance was observed when only the epidermis was input via the masked regression model (compare MAE $[S_{\text {nuclei}}]$ of 0.075 for regression and 0.081 for masked regression with no significant difference). This contradicts our hypothesis, as we observed a slight improvement when using the simple regression model, rather than the regression model with masked input. As the masked images have been validated with respect to semantic correctness, this suggests that the regression model does not need any additional pre-processing of the image and is able to infer a slightly superior approximation of $S_\text {nuclei}$ from the entire image. It is possible that the regression model even benefits from seeing tissue surrounding the epidermis, however, such supposition is hard to confirm, as the decision process of the regression model is handled intrinsically. Another interesting observation is that the regression model performs similarly well on both scores $S_\text {nuclei}$ and $S_\text {area}$. Though $S_\text {nuclei}$ and $S_\text {area}$ correlate, they do not perfectly match. One would expect a larger offset between these scores, if the regression model calculates the score similarly to a pathologist. It is possible that the regression model does not count cells to estimate $S_{nuclei}$, like pathologists do. Unlike the segmentation model and pathologists, the regression model outputs the score directly, where as pathologists and the segmentation model perform an intermediate step, counting entities such as cells or pixels before weighting them with a total of entities.

The $S_\text {area}$ score could be estimated with a regression model, but due to its inherent relation to segmentation, we choose to approximate $S_\text {area}$ with a segmentation model, where we compute the ratio of damaged areas and areas containing the epidermis based on the predicted segmentation. The Segmentation model performs much better on $S_\text {area}$ with an MAE of 0.052, than the regression model on $S_\text {nuclei}$ with an MAE of 0.075 and 0.081 respectively. When applying the estimated area scores of the segmentation model to $S_\text {nuclei}$, we observe a larger offset between respective MAE, with an MAE of 0.091 for $S_\text {nuclei}$. Still this offset is within a deviation respective to the MAE of 0.08 between these scores and to be expected, as we mentioned before the scores correlate but are not the same. We can observe that the best results are achieved when a model is not trained to learn predictions of scalar values, but rather taught semantic foundations, such as semantic segments, of individual terms (e.g. $A_\text {epi}$ and $A_{dmg}$) that model intermediate steps in the formulation of scalar output. That is if such prior knowledge of the formulation of the scores is present, as is for $S_{area}$ and $S_{nuclei}$. Regression models trained directly on $S_{area}$ yielded a mean and standard deviation of MAE of $0.078 \pm 0.003$ and $0.080 \pm 0.005$, for regular and masked regression respectively. Comparing to $S_{nuclei}$ yields mean and standard deviation of MAE of $0.091 \pm 0.002$. Thus we conclude that we do not compromise performance when predicting $S_{area}$ via an intermediate step over $A_\text {epi}$ and $A_{dmg}$. As a further remark on the difference in performance, when relating the scores $S_\text {area}$ and $S_\text {nuclei}$, we found that when ridding the data of samples where annotations of these scores seem uncorrelated the MAE of these scores drops significantly. We suspect that in the absence of noise, the correlation of the scores is higher than the proposed dataset suggests on surface level.

The segmentation approach delivers interpretable results by design which is the major advantage compared to the regression model where additional tools are needed to obtain semantic information. We experimented with three U-Net variations. We investigated the width of the U-Net model, required to yield good results on limited data. Smaller models can tend to generalize more and over-fit less on smaller datasets, but lack in complexity. To asses a good configuration of the U-Net, we have evaluated three variants: U-Net/16, U-Net/32 and U-Net/64 with respective base feature dimensions of 16, 32 and 64. All variants have been trained five times on each fold, results are listed in Table 2. We observe little improvement when exceeding a base feature size of 32 for most folds. The IoU scores of the models are close in general, however a lightweight U-Net/16 and U-Net/32 performs slightly worse than U-Net/64 on most folds, which suggests that its lack in complexity compromises prediction. We decide to use the U-Net/64 variant for the segmentation model, as it performed best with respect to IoU across folds and does not appear to be overfitting on the data.

### Practical relevance

When it comes to the application of a model in practice, many more measures of quality are desired, than just quantitative results of the MAE and IoU. Most of all robustness and interpretability are of essence for the practical relevance of a model. As discussed, on heterogeneous samples even pathologists tend to disagree on exact scores, see Fig. 1b. Therefore it is hard to assess the overall goodness of a model, when solely relying on the quantitative evaluation of scores. Especially for samples that may have been sampled differently, e.g. obtained by another lab or scanner, these quantitative results may not be representative for the models performance, due to the small amount of training data. A faithful application of such a model therefore has to accommodate some indefinite feedback, as to how and why it predicted a certain score. Given such additional information, the models predictive performance and sanity on unseen data can be validated on the fly by a pathologist on a subset of the unseen data.

The proposed regression models yield good quantitative results within the confides of similarly sampled data, but lack interpretability out-of-the box. That is to say explainability methods such as e.g. LRP^15,16^ have to be applied to infer some knowledge on relevant image area. Such explainability measures can indicate general areas of interest, but are not guaranteed to perfectly display relevant areas in great detail and can be misleading. While these methods help to identify general features or visual concepts, using them as means to explain how a regression model inferred the terms $N_\text {dmg}$ and $N_\text {all}$ of the score $S_\text {nuclei}$, where $N_\text {dmg}$ and $N_\text {all}$ denotes the number of damaged and all nuclei in the epidermis respectively, would be unreasonable. It is unclear if the regression model counts the nuclei at all. In particular, we investigated explanations for a random subset of samples and observed that the models contextualizes information from the environment of the epidermis in order the adjust predictions based on different amount and quality of staining. This observation is also supported by the fact, that masked regression does not benefit from excluding everything except epidermis.

**Figure 2:**
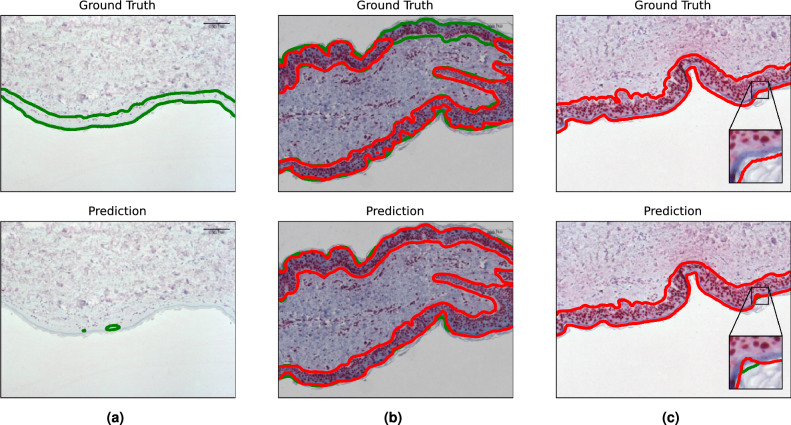
Qualitative examples from the segmentation model highlighting different aspects: (**a**) in case of low contrast, the performance drops considerably. (**b**) shows robustness to missing annotations (considered as label noise). Figure (**c**) shows a case where the metric (macro-average IoU) drops considerably (although qualitatively almost perfect) due to small predictions for absent classes (in this case for healthy epidermis (green) although completely damaged (red)).

The segmentation model provides a pixel-wise explanation of $A_\text {epi}$ and $A_\text {dmg}$, where $A_\text {dmg}$ is the number of pixels depicting damaged tissue and $A_\text {epi}$ the number of pixels depicting the epidermis. Not only can the surface area of semantic segments be inferred, but exact image locations can be visualized directly, allowing for direct feedback to the pathologist, without compromising predictive performance. Moreover, $S_\text {area}$ as a score itself tends to be less noisy, as can be observed form the standard deviation of predictions listed in Table 1. After a sample-level investigation of predictions, we found that a bad IoU score does not necessarily indicate a failed prediction. In Fig. 2b we observe that sample noise, though seldom, does occur for segmentation annotations as well. For these cases the model achieves a more accurate prediction than the noisy annotations themselves, hence inducing a noisy measurement of the IoU. In Fig. 2c we showcase a sample where the model gives an almost perfect prediction, with the exception of a small area, where healthy epidermis is predicted right next to the damaged segment. The ground truth annotation consists of damaged area only. This induces the IoU of this sample to drop significantly, as one class is predicted completely false and all classes are weighted equally. In this case, the macro-averaged IoU is more drastic than it needs to be to ensure a good score $S_\text {area}$. However, bad segmentation can coincide with accurate predictions of $S_\text {area}$. This case is visualized in Fig. 2a. The model fails to detect the epidermis, though to the lack of damage, the resulting score remains accurate. The sample in Fig. 2a, unlike most samples in the dataset, shows very low contrasts. The segmentation model appears to struggle with such low contrast samples, possibly related to these samples being underrepresented in training.

Finally, the robustness on out of distribution data, such as images sampled from another lab or scanner is hard to assess. To this day, state of the art models struggle to predict on out of distribution data, when trained or fine-tuned on relatively small datasets^17^. Hence, for applications where only small datasets are available human assessment of sanity and correctness is of essence, when applying a model to out of distribution data. The segmentation model allows for such assessment. By looking at the semantic segmentation of pixels contributing to the terms $A_\text {dmg}$ and $A_\text {all}$, a pathologist can quickly judge whether the model yields sane predictions on unseen data. Due to the importance of robustness and interpretability we recommend using the segmentation model and $S_\text {area}$ in practical application.

Practical applications include the evaluation of the protection of sunscreens, where DNA damages caused by UV light are counted in the epidermis. The challenge here are the inhomogeneously distributed damages, due to inhomogeneous distribution of sunscreen. The area model can evaluate the WSI, which is less biased compared to a human histopathologist who examines only a local region of interest in the WSI. Other application is the risk assessment of disinfection and inactivation of multi-resistant pathogens with UVC-radiation both on porcine skin^18^ and on human skin^14^. In order to inactivate multi-resistant pathogens like methicillin-resistant Staphylococcus aureus (MRSA) a UVC dose has to be chosen that inevitably leads to DNA damages in the epidermis and our model can support the histopathologist with the analysis of the DNA damages. For future work, the goal is to generalize the model to other stainings in the epidermis to extend the area of application.

## Discussion

In this work, we evaluate different approaches to estimate cell damage within the epidermis. We propose a new score $S_\text {area}$, based on segmentation labels of semantic segments that are contributing to cell damage in the epidermis. While we used related work in the field of deep learning for histopathology as a source for inspiration of applicable model architectures^6–9^, our scope in this work is to emphasize the need for different metrics and scores for reliable judgement of cell damage, rather than competing with related work on publicly available datasets. For this reason, we selected models which achieve satisfactory and robust results without excessive tuning of hyper-parameters. Since we deal with very high dimensional data, we need architectures with sufficient amount of complexity in order to minimize errors. This lead us to the selected models in this work, namely VGG16 with pretrained weights from ImageNet (omitting pretrained weights yields comparable results but at the cost of more epochs for convergence) for regression and vanilla U-Net for segmentation. For regression we observed that less complex and shallow model (like MobileNet or AlexNet) were not as capable as the VGG16, mostly due to too small receptive fields as compared to high dimensional input data. When fine-tuning other state of the art models, such as Inception, ResNet or transformer variants on the proposed dataset, we observed no significant gain in performance which would justify the additional amount of computational resources on such a small dataset. Moreover, to prevent overfitting, we replaced the classification head of VGG16 with less complex layers and dropout layers in between yielding reduced complexity.

The same observation holds for our vanilla U-Net for segmentation, where we reported three variants with increasing complexity yielding only marginal gains in performance, if at all. For this reason, we believe that our model selection hit a sweet spot of performance and complexity for the demonstration of different metrics and scoring methods.

Data were acquired in a collaborative development with medical professionals. Upon investigation of differences between annotations of different pathologists, we found that pathologists can vary a lot in their predictions on heterogeneous samples. Regarding possible external threats to validity, we already reduced selection bias by covering a broad range of different sources, amounts of cell damage and donors. Furthermore, we see potential in further decreasing this bias by considering an even more diverse set of training data by using different microscopes and sensors, which would induce a broader range of resolution and noise. The results obtained by our methods are competitive and in many cases better than clinical expectations with an MAE lower than 0.1. In fact, on a validation set which was labelled by two pathologists independently, we observed that the confidence intervals of our models’ error as compared to either pathologist is comparable or even below to those intervals among both pathologists. For this, we performed multiple statistical tests on one thousand bootstrapping samples for each scenario, were for each bootstrapping sample, we computed the mean absolute errors (visualized in Fig. 1d) and the standard deviation of absolute errors (visualized in Fig. 1e). We evaluated three scenarios: (1) among human pathologists (2) among the first pathologist (on whom the labels for training are based on) and our regression model and (3) among the second pathologist and our regression model. The first case shows a mean absolute error (MAE) of $\sim 0.23$ with a standard deviation of $\sim 0.24$ (see introduction and Fig. 1b). The second and third scenarios showing significantly lower means and standard deviations when considering the labels of both pathologists as ground truth respectively. Considering these observations and the accompanying increase in quality through segmentation, the performance of our models is comparable to that of human pathologists and thus suitable for real-world applications. Studies on interobserver variability among pathologists for different histological stains have reported varying levels of agreement^19,20^, indicating the need for standardized software tools in interpreting histological stains.

By exploiting the potential of semantic segmentation, we obtain a model with outputs that are directly interpretable with respect to $S_\text {area}$, without compromising predictive performance when compared to regression models.

As both, the regression and the segmentation model yield good quantitative results, we deem them as applicable to many tasks concerning cell damage in the epidermis. For clinical applications, where one relies on high accuracy and sanity of the output, we strongly recommend using the $S_\text {area}$ score of the segmentation model. The interpretability of this models output semantic segments allows for an understanding of whether the model performs well on unseen data or, if not, on what samples it lacks robustness.

as a final remark, having a pathologist annotate samples, whether it be $S_\text {nuclei}$ or $S_\text {area}$ is a costly process. One way to reduce such cost is an iterative process, similar to strategies used in active learning, where, from a pool of unlabeled data, samples with the most uncertainty are queried. Additionally, if similar domain-specific datasets with semantic segmentation are made public, transfer-learning could greatly improve the models performance as well.

In future works one could investigate the effects of various ensembles, as well as heavily augmented training data on this small dataset. The purpose of this work is a simple, straight forward baseline of what is to expect when dealing with different approaches to estimate cell-damage, namely counting nuclei ($S_\text {nuclei}$) and measuring the ratio of damaged areas to healthy areas in the epidermis ($S_\text {area}$).

## Materials and methods

### Data acquisition and labels

Relative cell count is defined by $S_{nuclei}=\frac{N_{dmg}}{N_{all}}$, where $N_{dmg}$ is the number of damaged cells and $N_{all}$ is the total number of cells visible in the epidermis in a given image. Since the information about one image is condensed into a single number, we consider this label as *weak label* as the human costs are relatively low (more precisely: human effort was wasted because a pathologist counted the two types of cells, but only reported the ratio). The score $S_{nuclei}$ was annotated for all 804 samples and is therefore our main target which we want to regress and evaluate.

The score $S_\text {nuclei}$ is motivated by nuclei being visible, singular cell-components. Therefore, $S_\text {nuclei}$ strongly correlates with the relative amount of damaged cells in the slide. However, pathologists rely on a staining to identify damaged nuclei and for heterogeneous slides with varying damage, this may induce noisy labels, as opinions on cell damage may vary in regions where the staining fades and samples with heterogeneous damage in general. The correlation of pathologist scores in Fig. 1b emphasizes this problem on the proposed dataset. In an effort to obtain less noisy labels, segmentation maps of deeply stained regions within the epidermis were labelled. Thus a new score $S_\text {area}$ can be obtained from the ratio of damaged regions to healthy regions within the epidermis. For a subset of 202 samples we created pixel-wise annotations for both, the epidermis and damaged cells. Based on this segmentation map we derived $S_\text {area}=\frac{A_{dmg}}{A_{epi}}$, where $A_{dmg}$ is the area (number of pixels) with damaged cells and $A_{epi}$ is the area (number of pixels) containing the epidermis. While $S_\text {area}$ is again considered as weak labels, the segmentation map itself is considered as *strong* label, since the human costs are higher but this type of label allows for more fine-grained evaluation and provides more feedback to the user.

The retrospective analysis was performed on histological images recorded from previously obtained skin samples with varying amount of DNA damage as explained elsewhere^14^. The acquisition of the dataset was carried out by expert pathologists with extensive domain-knowledge. All annotations concerning the $S_\text {nuclei}$ score were carried out by the same pathologist, all annotations concerning segmentations and the resulting $S_\text {area}$ score were annotated under supervision of another pathologist. The dataset consists of 804 samples in total, of which 487 (60%) are from human skin, 269 (34%) from human skin model and 48 (6%) from ex-vivo porcine skin. 415 (52%) are stained with CPD, 317 (38%) with 64PP, 36 (5%) with Model African American (MAA) and 36 (5%) with Model Asian Caucasian (MAC). For 202 (25%) of the samples we have annotation for epidermis and high damage areas. All experimental protocols were approved by the ethics committee of the Charité – Universitätsmedizin Berlin (EA1/324/19) and were performed according to the declaration of Helsinki as revised in 2013. Informed written consent was given by all subjects. In Fig. 1a we show one example where we highlighted the epidermis and areas with high damage. All images are taken at the same resolution of $0.645 \frac{\mu m}{pixel}$ and size ($\texttt {1040}\times \texttt {1384}$ pixels corresponding to $\sim \texttt {670}\times \texttt {892}~\mu m\sim \texttt {0.624}~mm^2$).

**Figure 3:**
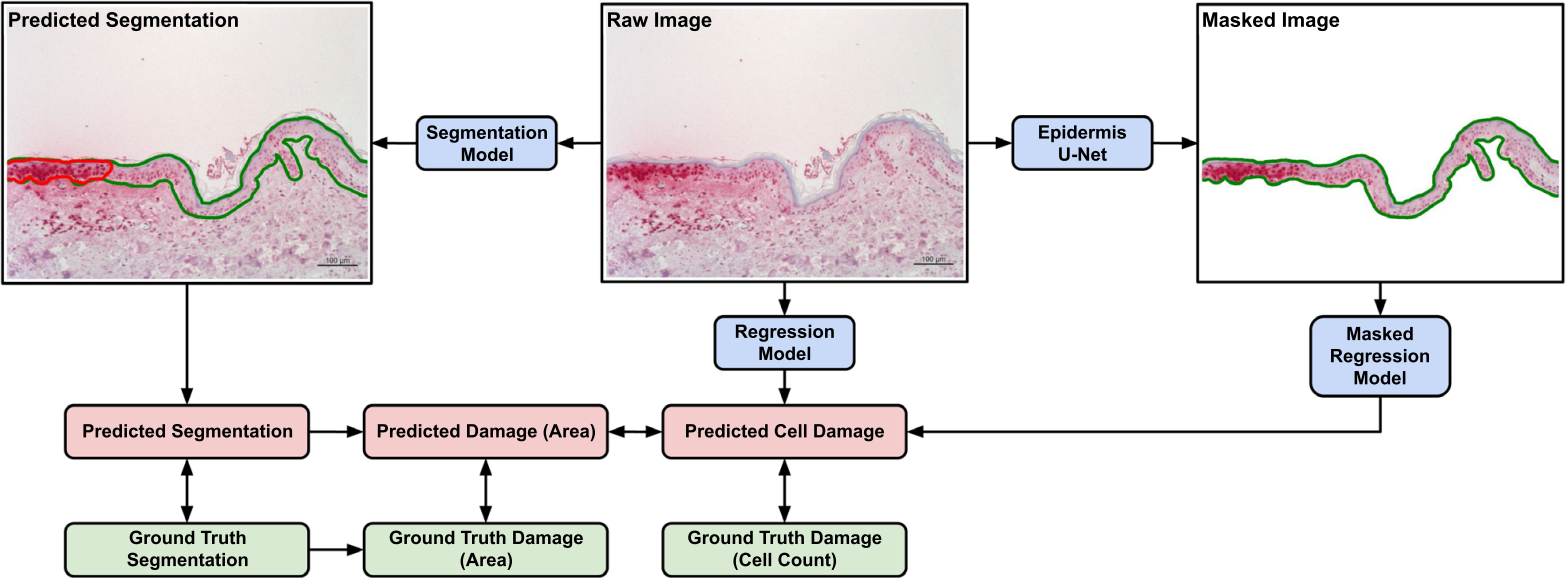
Overview of our proposed pipelines and methods for comparing different approaches exploiting different kind of labels. In order to guarantee comparability we also propose conversions between the different kind of outputs. While the *Regression Model* and *Segmentation Model* both operate directly on the image, the *Masked Regression Model* utilizes epidermis segmentation maps provided by a separately trained U-Net in advance.

To validate the sanity of $S_\text {nuclei}$ annotations, an additional pathologist labelled 18 critical samples with heterogeneous damage, resulting in an MAE of 0.23 between both. A respective scatter plot is depicted in Fig. 1b. The relation of the two scores $S_\text {nuclei}, S_\text {area}$ is depicted in Fig. 1c. Though only a small subset of nuclei were labelled by the additional pathologist, we observe strong deviation in heterogeneous samples. The score $S_\text {area}$, does correlate with $S_{nuclei}$ and though the distribution appears to be slightly skewed from the diagonal, we observe little drastic outliers. Hence we conclude $S_\text {area}$ to be the more robust measurement of damage.

Porcine ear skin is included in the data set because it is widely used in medical research as a suitable model for human skin^21,22^. Porcine ear skin and especially the epidermis exhibits many similarities like sparse body hair and epidermal thickness, structure and chemical properties compared to human skin^23^. The application of assessment of DNA damages in the epidermis was shown in porcine skin^18^, as well as human skin^14^.

### Animal ethics

Porcine ears were obtained from a local butcher. The donor pigs were six months old at the date of slaughter. The preparation of the porcine ears as well as the experiments took place at the day of slaughter. The experiments were authorized by the Commission of Consumer Protection and Agriculture, District Dahme-Spreewald, Germany. Porcine ears without any visible injuries were selected for further examinations.

### Experiments and metrics

To obtain sound quantitative results, we carried out a 10-fold cross evaluation. We configured stratified folds with the emphasis on the staining, tissue type and the presence of segmentation annotation for respective samples.

Though our evaluation is centered around the predictive performance with respect to the scores $S_\text {nuclei}$ and $S_\text {area}$, we are also concerned with the accuracy of the image areas $A_{dmg}$ and $A_{epi}$ on a spatial level. To ensure the correctness of $A_{dmg}$ and $A_{epi}$ is to ensure the correctness, sanity and interpretability of $S_\text {area}$, given a segmentation model for $A_{dmg}$ and $A_{epi}$. Therefore, in addition to the mean-average-error (MAE) for $S_\text {nuclei}$ and $S_\text {area}$, we decided to also report the IoU as a metric for the correctness of $A_{dmg}$ and $A_{epi}$ for the segmentation model. The IoU was measured by the Jaccard index for three classes: non-epidermis area, healthy epidermis and damaged epidermis.

### Data augmentation

We applied (1) random axis flips (horizontal and vertical) (2) random brightness adjustments sampled uniform between 0.75 and 1.25 and (3) random patch sampling for U-Nets with varying patch-sizes. During experimental analysis, we observed that all models performed reasonably within those limits and even slightly beyond. Although there are plenty of additional methods available for data augmentation^17^, we decided to focus on the most basic methods in order to keep the pipeline straight forward and efficient. In future studies we might include heavy augmentation in order to study effect occurring due to distribution shifts introduced by different staining, microscopes and tissue sources. However, given the small dataset at hand, the proposed simplistic augmentations yielded good results.

### Models

Both scores $S_\text {nuclei}$ and $S_\text {area}$ can be approximated with a regression model. Due to the annotations of the semantic segments, which are the individual terms contributing to the ratio $S_\text {area}$, we choose to exploit these annotations via a segmentation model, while relying on regression models for the $S_\text {nuclei}$ score. To disentangle the effects of tissue surrounding the epidermis for prediction we additionally evaluated a masked regression approach, where the regression model only receives image information concerning the epidermis. In Fig. 3 we provide an overview of our proposed methods involving different pipelines arriving at outputs keeping them comparable.

#### Regression and masked regression

For the standard regression models (plain and masked) we consider one whole image for inference. Since the original image of size $\texttt {1040}\times \texttt {1384}$ exceeds current capabilities of neural networks and memory of GPU’s, we downsampled the images by factor of 4 to $\texttt {260}\times \texttt {346}$. Tests with higher resolution images were conducted, but no gain in performance was noticed. On the contrary, the performance dropped, possible tied to the ratio of filter sizes and respective receptive fields to the input becoming very small with increasing input size. To avoid unnecessary complexity, we used lower resolution input and achieved competitive results.

The backbone of our regression models is a standard VGG16 model^24^, which was pre-trained on imagenet^25^ followed by a concatenation of global max and mean pooling (1024=512+512) and one additional ReLU layer (with 128 filters) followed by one output Sigmoid neuron. Though many complex models have conquered the state of the art in computer vision in recent years^26–28^, these approaches rely on large amounts of data to pre-train and fine-tune on. We opted for the well established VGG architecture, as it’s straight forward convolutions help the model to generalize on small datasets.

In addition to the regression on regular input images, we also investigated the effects of masking the image, eliminating image information that is assumed to be immaterial. After investigating the performance of the regular regression model, we noticed that the relevances (in terms of LRP maps^15^) were not located only in the epidermis (which is what we expect) but also in the periphery of the epidermis (inside and outside of the tissue). For this reason we trained a model similar to the regular regression model, but trained with masked epidermis images to enforce the model considering only pixels in the epidermis. To do so, we need information about the location of the epidermis in order to mask the image appropriately. Having a human mask the epidermis would defeat the purpose of the masked regression, hence a segmentation model was used to mask the images. We follow a basic U-Net^29^ architecture with four layer-blocks (32, 64, 128, 256 filters) for each the encoder and decoder (number of filters in reverse), where each layer consists of two convolutional layers two batchnorm layers and a maximum pooling operation, with residual connections to the decoders’ respective layer. We optimized the model using Adamax (with learning rate 0.001) minimizing binary crossentropy per pixel. We trained for 150 epochs, where in each epoch we sampled 50 batches, each consisting of 32 samples (i.e. 1600 patches per epoch). As training data we used $\texttt {256}\times \texttt {256}$ patches from $2\times$ down sampled images (corresponding to $\texttt {330}\times \texttt {330}~\mu m$).

#### Segmentation model

**Table 2:** MAE of the $S_{\text {area}}$ score and IoU scores of U-Net variants across all folds.

Fold	U-Net/16		U-Net/32		U-Net/64	
	MAE $[S_{area}]$ $\downarrow$	IoU $\uparrow$	MAE $[S_{area}]$ $\downarrow$	IoU $\uparrow$	MAE $[S_{area}]$ $\downarrow$	IoU $\uparrow$
1	0.031 ± 0.013	0.788 ± 0.020	**0.021** ± **0.006**	**0.815** ± **0.016**	0.026 ± 0.005	0.809 ± 0.028
2	0.051 ± 0.003	0.743 ± 0.015	**0.048** ± **0.008**	**0.774** ± **0.007**	0.054 ± 0.017	0.773 ± 0.008
3	0.065 ± 0.021	0.809 ± 0.011	0.073 ± 0.016	**0.816** ± **0.008**	**0.063** ± **0.037**	0.802 ± 0.015
4	0.055 ± 0.009	0.759 ± 0.015	0.052 ± 0.007	0.756 ± 0.018	**0.046 ± 0.008**	**0.762** ± **0.014**
5	**0.038** ± **0.004**	0.775 ± 0.008	0.057 ± 0.015	0.764 ± 0.025	0.041 ± 0.004	**0.786** ± **0.015**
6	0.037 ± 0.009	0.797 ± 0.021	0.040 ± 0.009	0.800 ± 0.028	**0.037** ± **0.007**	**0.811** ± **0.021**
7	**0.045** ± **0.005**	0.724 ± 0.014	0.048 ± 0.006	0.736 ± 0.018	0.046 ± 0.009	**0.766** ± **0.016**
8	**0.018** ± **0.017**	0.795 ± 0.033	0.019 ± 0.007	0.789 ± 0.017	0.020 ± 0.009	**0.807** ± **0.018**
9	0.058 ± 0.009	0.726 ± 0.002	**0.046** ± **0.005**	0.743 ± 0.013	0.049 ± 0.005	**0.748** ± **0.020**
10	0.017 ± 0.006	0.825 ± 0.016	0.019 ± 0.007	0.843 ± 0.024	**0.014** ± **0.005**	** 0.866** ± **0.043**
All	0.045 ± 0.006	0.764 ± 0.004	0.047 ± 0.005	0.776 ± 0.004	**0.044** ± **0.004**	**0.785** ± **0.003**

Significant values are in [bold].

Though the score $S_\text {area}$ could be estimated by a regression model in a similar manner as $S_\text {nuclei}$, we deliberately choose to estimate the individual terms $A_{dmg}$ and $A_{epi}$ of $S_\text {nuclei}$, rather than directly the score. To estimate the semantic segmentation maps of high damage areas within the epidermis, as well as the epidermis itself, we resorted to a U-Net as the backbone of our segmentation model. Segmentation models such as the U-Net^29^ usually assume input within a certain range of resolutions. The images of the proposed dataset have a resolution of $\texttt {1024} \times \texttt {1344}$. To estimate segmentation maps on these images, a sliding window approach is used, predicting for each window frame and finally inferring from the collection of predictions. However, with respect to large input images, the U-Nets receptive field is limited if filter sizes are not increased. We decided to keep the filter size constant and adjusted the input size via down-sampling. Images were downsampled by a factor of two. We used a U-Net that pools three times, for input image-patches at a scale of $\texttt {256} \times \texttt {256}$. Three model variants U-Net/16, U-Net/32 and U-Net/64 were investigated and evaluated. We observed that the U-Net/64, that has a base feature dimension of 64 performed best, though increasing the base feature dimension does not yield great performance enhancements beyond U-Net/32. Results are listed in Table 2.

All U-Net variants were trained for 50 epochs with 100 steps per epoch and a batch size of 32. We used AdaMax with categorical cross entropy, a learning rate of 0.001 and hyperparameters $\beta _1=0.9, \beta _2=0.999, \epsilon =10^{-7}$. The training was kept consistent to ensure a fair comparison of variants. Batch sizes larger than 32 did not yield better results, possibly due to the size of the dataset. To virtually increase the variance of the training data, we augmented the training data, using random brightness adjustments within $[75\%, 125\%]$, random vertical and horizontal flips, as well as random rotations within $[-15^\circ , 15^\circ ]$.

## Data Availability

This work was partly funded by the German Federal Ministry for Education and Research as Patho234 (ref. 031LO207). The datasets generated and/or analyzed during the current study are available in the zenodo repository https://doi.org/10.5281/zenodo.7282326. All samples were anonymized and processed in accordance with the institutional guidelines and cannot be traced back to an individual person. All code related to this work is available upon request from the corresponding author.
